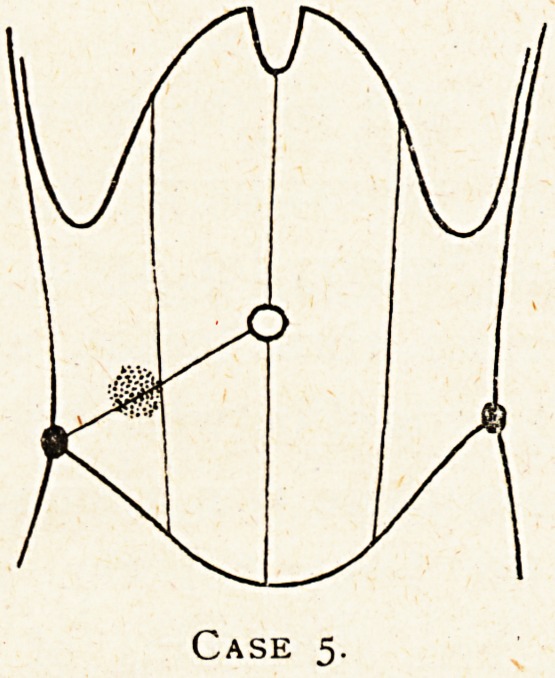# The Differential Diagnosis of Malignant Disease of the Cæcum from Chronic and Subacute Appendicitis

**Published:** 1922-09

**Authors:** Charles A. Morton

**Affiliations:** Professor of Systematic Surgery in the University of Bristol; Consulting Surgeon to the General Hospital and the Children's Hospital; Examiner in Surgery in the University of Birmingham


### THE DIFFERENTIAL DIAGNOSIS OF MALIGNANT DISEASE OF THE CÆCUM FROM CHRONIC AND SUBACUTE APPENDICITIS.

BY

Charles A. Morton, F.R.C.S.,

*Professor of Systematic Surgery in the University of Bristol; Consulting Surgeon to the General Hospital and the Children's Hospital; Examiner in Surgery in the University of Birmingham.*

It is sometimes impossible to make a definite diagnosis between a malignant growth of the cæcum, and a chronic, or subacute, appendicitis. These case records will, I think, illustrate this difficulty, and will show how puzzling the signs and symptoms may be. Even on opening the abdomen the difficulty in distinguishing the one condition from the other may still be marked. This was so in all the three malignant cases (2, 3 and 5). The condition of the mass found within the abdomen in these cases might have been the result of adhesive appendicitis, with inflammatory thickening of the adjacent cæcal wall, with or without pus enclosed by adhesions, or to malignant growth, and it was not until the adhesions had to a certain extent been broken down that it was possible to make a certain diagnosis.

The almost complete absence of pain in chronic appendicitis, even with abscess formation, is very striking in the record of Case 1, and the intense hardness with nodulation of an inflammatory appendix swelling in Case 4. On the other hand, the way in which a case of malignant disease may simulate an attack of appendicitis with marked pyrexia is well shown by the record of Case 5. In all the cases, both inflammatory and malignant, the lump was quite fixed in the right iliac fossa, and was situated where the swelling due to appendicitis is very often found. In one of the malignant cases (Case 5) it was at times distinctly tender. On the other hand, a swelling due to appendicitis, even when suppuration is present, may at a certain stage be quite free from tenderness, and the pain from which the patient has suffered may have completely subsided, and no pyrexia may be present, even in the evenings.

In one of the cases (Case 3) there was both malignant growth and appendicitis, the latter condition being due to blockage of the lumen of the appendix by the growth.

**Case 1.—A case of chronic appendix abscess simulating malignant disease of the cæcum.**

Mrs. M., aged 74, was seen in consultation with Dr. Crossman on August 29th, 1912, with a swelling in the right lower abdomen. She had noticed it accidentally three months before I saw her, and there had been some slight shooting pain in it. During the three months there had been occasional vomiting. She had been no more constipated during that period than was habitual to her. She had been seen by Dr. Crossman a week before we saw her together for vague pains in various parts of her body, and at that time he had found her temperature was raised to between 100° and 101°. The patient only mentioned the presence of the lump some days later. Her temperature was then found to be raised to the same extent as on the previous occasion.

The abdominal wall was quite thin, and there was visible fulness in the right lower abdomen. There was a very definite hard lump (A) just inside and below the anterior superior spine (C) of the size and shape of a thin thumb beyond the last joint; above it was an ill-defined mass, of which I could not define the upper border (B). Both the well-defined lump A and the more diffused swelling B were fixed. There was no general abdominal distension and no evidence of free fluid. There was nothing further to be made out on a pelvic examination.

There seemed to have been remarkably little pain for a chronic appendicitis. When Dr. Crossman had attended her for the vague general pains a week before we saw her together she did not even then draw attention to the abdominal lump. The very moderately raised temperature was rather suggestive of appendicitis, but on the other hand is often present at times in malignant disease of the intestine. The lump A felt quite definite enough to be a nodule of growth. The more diffuse swelling B felt like matted intestine. It was resonant. The diagnosis between chronic appendicitis and malignant growth seemed uncertain.

I operated in the General Hospital on August 31st, and found an appendix abscess under the head of the cæcum. The whole cæcum was buried under a mass of adherent omentum, which fixed it to the parietal peritoneum. I was able to remove the diseased appendix, and the patient made a good recovery. The nodule A must have been a piece of thickened adherent omentum. It is interesting that this abscess should have remained so quiescent for at least three months. It is, of course, possible that the lump the patient then noticed was due to a chronic appendicitis which had not at that time suppurated, but there had been no acute symptoms supervening since which would suggest its onset at a later period.

**Case 2.—A case of malignant growth of the cæcum in which the diagnosis from chronic appendicitis was difficult.**

Louisa W., 41, was admitted to the General Hospital on January 4th, 1907, on the recommendation of a doctor who having made a general examination of her chest and abdomen had discovered a lump in her right lower abdomen, but she had had no pain there, and was quite unaware of the existence of the swelling. She consulted her doctor for chest trouble. At the position shown on the diagram there was a round, very hard, fixed swelling the size of a hen's egg, which was not tender. There had been no abnormal condition of the bowels. It was not possible to say if it was chronic appendicitis or new growth. The complete absence of pain was certainly in favour of the latter. Under anæsthesia just before the operation was commenced I could push the lump well inwards towards the pelvic brim, but neither up nor down at all.

I opened the abdomen on January 8th, and found the cæcum and appendix were fixed to the parietal peritoneum by firm adhesions, which also fixed the whole appendix to the cæcum. It was at first doubtful if the swelling was part of a chronic appendicitis, but after separating the adherent appendix, it seemed probable that it was cæcal growth. Some enlarged

Case 1.

Case 2.

glands under the peritoneum on the inner side of the cæcum confirmed this view. I finally opened the cæcum and examined the interior with my finger to make quite sure. I then excised the cæcum and several inches of the lower ileum, and united the ileum and colon. The growth, which was of a dense, white character and ulcerated, was situated around the ileo-colic junction and involved, but did not appear to block, the orifice of the appendix. The ileo-cæcal orifice was much contracted, and in the lower four inches of the ileum the muscle coat was much hypertrophied. There had evidently been partial obstruction in the ileo-colic junction, though no symptoms had been produced. The distal part of the appendix was also much thickened from hypertrophy of its muscle coat, due apparently to obstruction by kinking from adhesions, for there was no stricture within its lumen, and as already stated the orifice, though surrounded by the new growth, was not obstructed by it.

The patient was considerably "shocked" after the operation, and became even more so some hours later, but improved after intravenous saline infusion. She died the next day, with symptoms of obstruction of the small intestine, but there was no post-mortem examination.

**Case 3.—A case of malignant growth of the cæcum with secondary appendicitis.**

E. P., a man aged 58, was admitted as a case of appendicitis from the medical out-patient department on August 4th, 1914, in which he was first seen on that day. The history was of a week's increasing pain in the right lower abdomen, which had not laid him up. He had had no pain there before that. There had been no vomiting. He had been troubled with constipation for some months, but the bowels had been well open during the day or two before he came to the hospital. There had been no passage of blood or mucus from the bowel.

At MacBurney's spot (see diagram) there was a firm, well-defined, slightly tender lump the size of a hen's egg. There were no evening rises of temperature. The short history of the pain and its increase during this short period was suggestive rather of a subacute appendicitis, but the lump felt very much as if it might turn out to be new growth, and the absence of any evening temperature was rather in favour of growth, but of course was quite possible with a subacute appendicitis or even an appendix abscess.

I operated on August 5th, and found the end of the cæcum involved in a hard mass, which also involved the proximal end of the appendix, the distal end of which was red and swollen. Thus it was evidently an appendicitis, but it was not at first possible to be sure whether the lump in the cæcum at the base of the appendix was also inflammatory. Some exudation fixing the parts to the iliac fossa seemed rather excessive for malignant growth adhesion. In order to try and decide the question, after packing off the peritoneal cavity I endeavoured to separate the proximal portion of the appendix from the cæcum, but at once broke into the latter, and then on passing my finger into its interior I could feel a protruding mass of growth within it, which on examination after removal was found to be a protruding, nodular, sloughy growth attached to the wall in the region of the ileo-cæcal junction, but involving the base of the appendix, and quite blocking its lumen. Microscopic examination showed it to be a typical adenoid cancer. I excised the cæcum, ascending colon, and lower ileum, and the patient made a good recovery. Four years later there was an extensive recurrence in the abdominal wall in the region of the operation scar, but no recurrence within the abdomen. I excised the recurrent growth. When seen two years later there was no signs or symptoms suggesting any further recurrence either in the abdominal wall or in the abdomen.

**Case 4.—A case of appendicitis somewhat suggestive of the possibility of new growth.**

Walter L., 50, was brought to me at the General Hospital on May 29th, 1920, by Dr. Macvicker of Street, with a history that he began to suffer from pain across the upper abdomen a fortnight before, but had continued at work for the first week, and was then laid up for three days, and then resumed work again, but had been again obliged to give up the day before his admission, and in the evening of that day the pain had been marked. It had passed from the upper abdomen into the right lower abdomen. There had been no vomiting, and a tendency to constipation had been dealt with by the administration of an

CASE 3.

CASE 4.

aperient. On admission he had very little pain, and normal temperature and a pulse of 80. There was a very hard and tender lump in the position shown in the diagram, and when examined under the anæsthetic just before operating it felt so hard as to suggest it might be a tuberculous nodule or new growth. The abdomen elsewhere was normal. The marked tenderness of the lump, and the position of the initial pain and its marked increase in the evening before admission after it had passed to his right lower abdomen, certainly were suggestive of appendicitis, but the previous history that the onset had been subacute, and that he had been able to continue at work, together with the hardness of the lump, were rather suggestive of new growth.

I operated on the day of admission. A little clear serous fluid escaped on opening the abdomen, and on palpating the mass it felt even harder and more nodular than it had on examination through the abdominal wall under the anæsthetic.

This condition was due to the very marked thickening of the meso-appendix, and its projection, together with the indurated tip of the appendix, from a mass formed by fusion of the appendix and the meso-appendix with the indurated head of the cæcum. There was a little pus shut up by adhesions under the cæcum, and on separating the appendix from the cæcum pus was seen leaking out of a perforation about its middle. The cæcal wall was very considerably indurated. No omentum was involved in the mass, it was due entirely to marked inflammatory thickening of the cæcum, appendix, and meso-appendix. After removal the perforation of the appendix was found to be in an eroded patch of mucous membrane, but there was no sloughing. There was a small concretion, but its connection with the erosion and perforation was doubtful. The patient made a good recovery.

**Case 5.—A case of malignant disease of the base of the cæcum simulating subacute appendicitis.**

Mr. D., 57, was seen in consultation with Dr. Willcox of Glastonbury on July 29th, 1921. The history was that three months before I saw him he had been laid up for a few days with a febrile attack, and during this attack he had some abdominal discomfort, and Dr. Willcox then discovered a swelling in the right lower abdomen, which however disappeared after a free action of the bowels following the administration of an aperient. There was then no recurrence of any abdominal discomfort for more than two months, and no indication for any further abdominal examination, but on July 16th he began to get some discomfort in the right lower abdomen. If it was a definite pain it was certainly never a marked one. With this abdominal discomfort he ran a temperature of 102° in the evening and 100° in the morning for five days. Dr. Willcox again discovered a swelling in the right lower abdomen, and found it distinctly tender, and so it remained during the period of the pyrexia. When I saw him on July 29th he had recovered from this attack, and had been about again for nearly a week without any abdominal discomfort on slight exertion. Dr. Willcox thought the lump was smaller, and it had ceased to be tender. There had been no difficulty in getting the bowels to act freely, indeed for a few days they had acted too freely.

On examination of the abdomen I found a very considerable thickness of fat in the abdominal wall, so that it was not at first easy to discover the swelling, but on deep pressure I found it. It was hard and round and quite fixed, of the size of a small hen's egg and not at all tender, and lay in the position shown in the diagram. He had been abroad during the war and then had dysentery and typhoid. The facts in favour of chronic appendicitis were the complete disappearance of all abdominal discomfort for two and a half months, the marked pyrexial nature of at any rate the second attack, and the distinct tenderness of the swelling in this attack, but perhaps most of all the disappearance of the lump after the first attack. Looking back at the case in the light of the operative finding, it is evident it did not really disappear. It was at all times difficult to palpate because of the amount of fat in the abdominal wall, and it might easily have been buried by distention of the bowel. In favour of new growth was the very slight amount, if any, of actual pain experienced ; and it is, of course, well known that patients with malignant disease of the bowel may get periods of pyrexia. In Case 1 I had found even with an appendix abscess almost no pain, and this encouraged me to hope the swelling was due to chronic appendicitis. After I saw him the recurrence of tenderness and the presence of rigidity of the abdominal wall made it impossible to feel the lump for a day or two. When again palpable it was larger.

CASE 5.

I operated on August 14th. Under the anæsthetic the lump felt very definite and hard. On opening the abdomen, the upper part of the cæcum seemed normal, but its base was fixed to the iliac fossa by an indurated mass—just as if it had been set on a hard bed of plaster of Paris—and in this mass the appendix was lost. There were no enlarged glands and no secondary growths in the liver. I thought it might turn out to be a subcæcal appendix abscess with great induration around, and I proceeded to separate the base of the cæcum from the iliac fossa, but as I did so, and was able to grasp the partially separated cæcum between my finger and thumb, I could feel a nodular growth projecting into its lumen from the base, and in the line of separation I found growth infiltrating the iliac muscle. I think, perhaps, it would have been wiser to have abandoned any further operation at this stage, but I hoped to be able to dissect away the infiltrated muscle, or if I could not wholly do so to deal with it later by radium or X-rays, and I therefore proceeded to excise the cæcum and ascending colon and united the ileum to the transverse colon. An examination of the parts removed at the operation revealed a nodular ulcerating growth encircling the base of the cæcum, and in it the appendix was lost. It had not obstructed the ileo-cæcal opening. After the excision of the cæcum and union of ileum and colon he was not in a condition of serious shock, but it was doutbful if he would bear any extension of the operation, and therefore no attempt could be made to remove the infiltrated muscle.

Signs of extensive infection of the wound became evident by the third day after the operation, but no signs of any infection of the peritoneal cavity. On the second day after the operation the disproportion between the frequency of the pulse and the heart's action became very marked, but with frequent injections of strychnine and strophanthin at Dr. Willcox's suggestion the condition passed off, but on the fifth day it steadily failed, and he died.

It may be well to add to the records of these cases a word as to carcinoma originating in the appendix itself. In the *British Journal of Surgery* for April, 1921, p. 392, Wilkie of Edinburgh describes three cases, and states that the literature contains records of between two and three hundred. In Wilkie's three cases there was a small nodule of growth which obstructed the lumen of the appendix, and thus produced very marked symptoms of appendicitis. This growth presented the microscopic structure of alveolar carcinoma, but there is no evidence that it was really a malignant growth. All three patients were under 23 years of age. Several writers have expressed the view that the nodules of the growth in the appendix in these cases are not really malignant. Wilkie himself expresses the opinion that "at present we must regard primary growths of the appendix as being of a relatively benign character, and liable to threaten life more from the acute obstruction and inflammatory complications to which they may lead than from any intrinsically malignant properties."

## Figures and Tables

**Case 1. f1:**
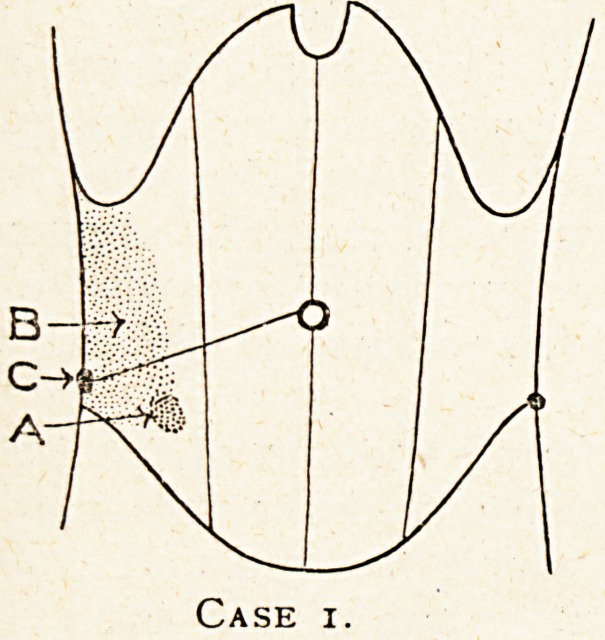


**Case 2. f2:**
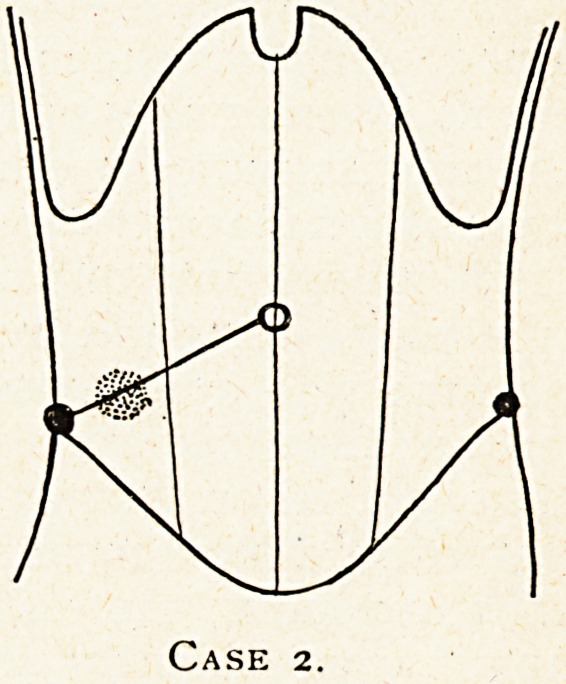


**Case 3. f3:**
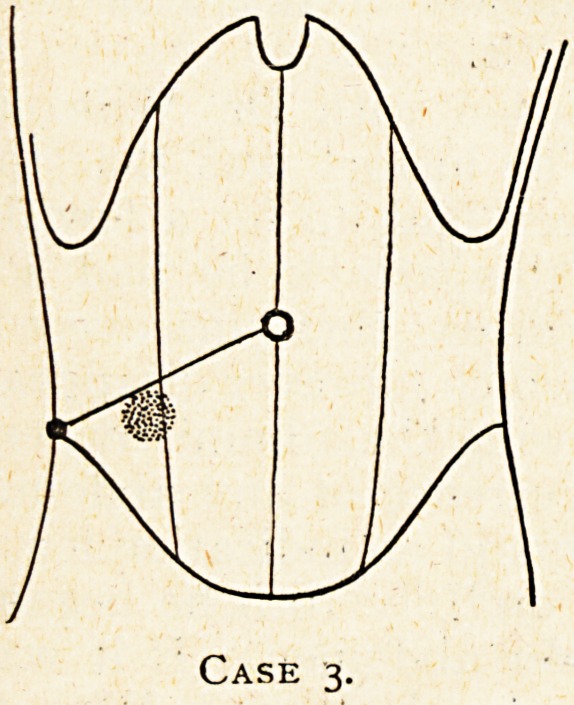


**Case 4. f4:**
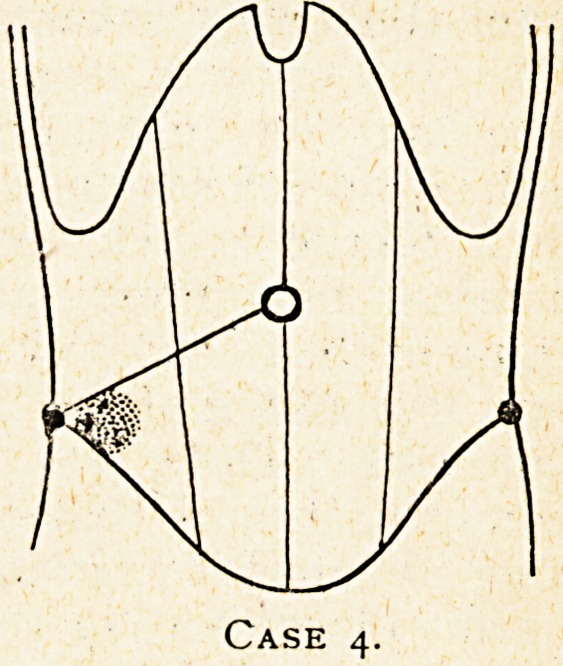


**Case 5. f5:**